# *SCN5A* U12‐Type Minor Intron Variant Causes Cryptic Splicing and Cardiac Arrhythmia Disease

**DOI:** 10.1155/humu/4537601

**Published:** 2026-09-24

**Authors:** Emma S. Singer, Serena Li, Ginell N. Ranpura, Seakcheng Lim, Sean Lal, Adam M. Bournazos, Sandra T. Cooper, Zachary Laksman, Richard D. Bagnall

**Affiliations:** ^1^ Bioinformatics and Molecular Genetics Group at Centenary Institute, The University of Sydney, Sydney, New South Wales, Australia, sydney.edu.au; ^2^ Faculty of Medicine and Health, The University of Sydney, Sydney, New South Wales, Australia, sydney.edu.au; ^3^ Molecular Cardiology Program at Centenary Institute, The University of Sydney, Sydney, New South Wales, Australia, sydney.edu.au; ^4^ Kids Neuroscience Centre, Children′s Hospital at Westmead, Westmead, New South Wales, Australia; ^5^ Neuromuscular Gene Discovery Group, Children′s Medical Research Institute, Westmead, New South Wales, Australia; ^6^ Centre for Heart Lung Innovation, University of British Columbia, Vancouver, Canada, ubc.ca; ^7^ Department of Medicine and the School of Biomedical Engineering, University of British Columbia, Vancouver, Canada, ubc.ca

**Keywords:** Brugada syndrome, genetic heart disease, induced pluripotent stem cells, minor introns, RNA splicing

## Abstract

Variants affecting minor (U12‐type) intron splice sites are underreported, and their consequences are poorly understood, which makes predicting their pathogenicity challenging. We investigated the impact on splicing of an *SCN5A* minor intron donor variant in an individual with cardiac arrhythmias using patient‐specific induced pluripotent stem cell cardiomyocytes (iPSC‐CMs). Genetic testing in the proband identified an *SCN5A* c.392+3A>G variant of uncertain significance in a minor intron donor splice site, which segregated in affected family members with cardiac phenotypes, including Brugada syndrome. Transcriptome sequencing of proband‐specific iPSC‐CMs, treated with and without inhibition of nonsense‐mediated decay, revealed aberrant *SCN5A* splicing. Four variant‐associated isoforms were absent from control iPSC‐CMs and control myectomy samples, and one skipped Exon 3 with a 96 bp extension of Exon 4. All alternatively spliced *SCN5A* transcripts were T/A cloned and confirmed by Sanger sequencing. These alternatively spliced transcripts used novel combinations of cryptic minor and major splice sites and introduced premature termination codons or an in‐frame deletion, supporting reclassification of the variant as likely pathogenic. A genome‐wide analysis of SpliceAI scores across minor intron splice sites retrieved from the Minor Intron Database revealed clear patterns of variants with high predicted splicing impact. This analysis also highlighted distinct splice site sequence motifs between minor and major splice sites that are not currently considered in ACMG/AMP variant interpretation guidelines. We further demonstrate that some cryptic donor–acceptor motif pairings are likely incompatible with splicing, which may inform future improvements in in silico splice site prediction tools. Integrating functional RNA sequencing with SpliceAI‐based predictions at minor introns offers a pathway towards refining variant interpretation frameworks in clinical genomics.

## 1. Introduction

Pathogenic variants in the sodium voltage‐gated channel *α*‐subunit 5 gene, *SCN5A* (MIM #600163), cause long QT syndrome type 3 (LQTS3), Brugada syndrome (BrS) and cardiac conduction disease [1]. Collectively, these primary cardiac arrhythmia syndromes affect up to 1 in 2000 individuals and can present with overlapping clinical features, including palpitations, syncope, ventricular fibrillation and sudden cardiac death [1]. The SCN5A protein regulates the influx of sodium ions (*I*_Na_) into cardiomyocytes to initiate the cardiac action potential, and disruption of SCN5A alters *I*_Na_ balance through either gain‐ or loss‐of‐function mechanisms [2, 3]. Gain‐of‐function variants lead to excessive *I*_Na_ and prolonged repolarisation, causing LQTS3, whereas loss‐of‐function variants lead to reduced *I*_Na_ and underlie BrS and cardiac conduction disease [4–6]. Pathogenic *SCN5A* variants also cause dilated cardiomyopathy through both gain‐ and loss‐of‐function mechanisms [3, 7].

An important class of loss‐of‐function variants disrupt RNA splicing, the posttranscriptional processing of pre‐mRNA that removes introns and joins exons to produce mature mRNA. Most introns are spliced out by the major spliceosome, which recognises ‘GT‐AG’ introns defined by almost invariant 5 ^′^ GT and 3 ^′^ AG terminal dinucleotides and a conserved branchpoint adenosine upstream of the 3 ^′^ end (Figure S1A) [8]. These introns are termed ‘major’ or ‘U2‐type’ introns as they are recognised by the U2 small nuclear ribonucleoprotein within the major spliceosome [9]. A small subset of human introns (~0.5%), found in approximately 700 genes, are termed ‘minor’ or ‘U12‐type’ introns. These typically have ‘AT‐AC’ or ‘GT‐AG’ terminal dinucleotides and differ from U2‐type introns by having distinct consensus donor and acceptor sequences and a more defined branchpoint near the 3 ^′^ end (Figure S1B) [9–13].

Variants in the canonical splice sites or branchpoints of major introns can cause exon skipping, intron retention or cryptic splicing at alternative GT‐AG dinucleotides. However, the functional impact of variants in minor introns is not as well characterised. Possible consequences include activation of alternative splicing at minor or major splice sites, or a combination of both sites, exon skipping or intron retention. Furthermore, in silico tools that predict whether a variant alters splice site strength are less reliable for minor introns due to limited training data for machine learning approaches, and in some tools, such as MaxEntScan, minor introns have been excluded from the design altogether [14].

Over 500 genes expressed in the human heart contain at least one minor intron, including *SCN5A* isoform NM_000335.5, which has a minor intron between Exons 3 and 4 [10, 15]. Knockdown of an essential minor spliceosome component in neonatal rat ventricular myocytes led to retention of this intron, reduced *SCN5A* protein expression and decreased *I*_Na_ current [15]. However, the effects of sequence variants in the essential splice sites of this minor intron in human heart tissue with an intact minor spliceosome are unknown.

Here, we report a family with cardiomyopathy and arrhythmias with an *SCN5A* minor intron variant of uncertain significance (VUS), NM_000335.5: c.392+3A>G. Analysis of RNA extracted from peripheral blood of the proband was uninformative due to very low *SCN5A* expression. Therefore, we used patient‐specific induced pluripotent stem cell cardiomyocytes (iPSC‐CMs) as surrogate heart tissue to assess splicing outcomes. Additionally, we performed a comprehensive analysis of sequence conservation and in silico predictions of splicing disruption across all human minor introns using SpliceAI [16] to identify essential regions within this understudied intron subgroup.

## 2. Materials and Methods

### 2.1. Clinical Evaluations

The proband, her brother and their parents were recruited to the British Columbia Inherited Arrhythmia Program at St. Paul′s Hospital in Vancouver, Canada. Family members underwent clinical evaluations including electrocardiography (ECG), exercise ECG treadmill testing, Holter monitoring, echocardiograms, sodium channel provocation testing and cardiac magnetic resonance imaging.

### 2.2. Genetic Testing

Exome‐based sequencing of a 184‐gene comprehensive cardiology panel was performed at Blueprint Genetics (Espoo, Finland), and only variants deemed to be clinically relevant were reported. Additional variant evaluation was performed using four in silico splice prediction tools: MaxEntScan [14], AdaBoost and random forest [17] and SpliceAI [16]. Loss of the donor or acceptor site was predicted by a reduction ≥ 4 in the MaxEntScan score, while the gain of a new splice site was predicted with an increase ≥ 4 in MaxEntScan score. A score > 0.6 with AdaBoost and random forest, or SpliceAI *Δ* score > 0.5, predicted a disruption to the splice site, as recommended by the developers.

### 2.3. Segregation Analysis

The *SCN5A* variant was genotyped in the proband, brother, father and mother (Macrogen, Inc., South Korea). PCR and sequencing primers were designed using Primer3Plus (https://www.primer3plus.com/index.html) (Table S1). Chromatograms were analysed using Sequencher v.5.4.6 (Gene Codes, Michigan, United States).

### 2.4. Generation of iPSC‐CMs

Peripheral blood mononuclear cells of the proband (II:2) were reprogrammed into induced pluripotent stem cells and characterised (https://hpscreg.eu/cell-line/CIAUi002-C) [18]. iPSC‐CMs were derived from the proband in triplicate and three unrelated control females using published protocols [19]. iPSC‐CMs were cultured in a 12‐well plate for up to 34 days at 37°C with 5% CO_2_, prior to 24‐h culture in media supplemented with 50 *μ*g/mL cycloheximide (Sigma‐Aldrich, Massachusetts, United States) to inhibit nonsense‐mediated decay and enable detection of transcripts potentially degraded by this pathway, or 1 *μ*L/mL DMSO vehicle control, prior to RNA extraction.

### 2.5. RNA Extraction

Snap‐frozen myectomy tissue samples from three unrelated female donor hearts were obtained from the Sydney Heart Bank (Table S2) [20]. RNA was extracted from iPSC‐CMs and 20 *μ*g snap‐frozen myectomy tissue using 1 mL of TRIzol Reagent (Invitrogen, Massachusetts, United States), as previously described [21].

### 2.6. Transcriptome Sequencing

RNA integrity was determined using an Agilent 2100 Bioanalyzer with the RNA 6000 Nano Kit (Agilent Technologies, California, United States) according to the manufacturer′s protocol. Samples suitable for transcriptome sequencing required an RNA integrity number > 7.0. Transcriptome sequencing libraries were prepared using the TruSeq Stranded mRNA‐seq kit (Illumina, California, United States), which underwent 101 base pair (bp) paired‐end sequencing on a NovaSeq 6000 Platform (Illumina) at the Ramaciotti Centre for Genomics, University of New South Wales, Sydney, Australia. Sequence reads were aligned to the GRCh38 genome reference using STAR and *SCN5A* splicing was visualised using the Integrated Genomics Viewer, as previously described in detail [19]. Sashimi plots of alternative splicing were produced using the Sashimi_plot package of Miso following the developers′ instructions [22].

### 2.7. T/A Cloning of Reverse Transcription PCR Products

Primers were designed using Primer3Plus to RT‐PCR amplify alternatively spliced *SCN5A* transcripts, as previously described (Table S1) [21]. Amplification products were agarose gel‐resolved and purified using the QIAquick Gel Extraction Kit, according to the manufacturer′s instructions (Qiagen, Hilden, Germany). Gel purified PCR products were cloned into the pGEM‐T Easy Vector System (Promega, Wisconsin, United States) at a concentration ratio of 3:1. Two microlitres of each ligation reaction was transformed into 50 *μ*L of One Shot TOP10 Chemically Competent *E. coli* (Invitrogen) and plated on LB agar, Lennox plates (BD Biosciences, New Jersey, United States) with 100 *μ*g/mL ampicillin (Sigma‐Aldrich), 0.5 mM IPTG (Promega) and 80 *μ*g/mL X‐gal (Promega) for blue–white colony selection. Positive colonies were picked and cultured overnight in LB Broth, Lennox (BD Biosciences), and plasmid DNA was purified using the QIAprep Spin Miniprep Kit (Qiagen) in 50 *μ*L elution buffer. Purified plasmid DNA was Sanger sequenced at Macrogen, Inc. (South Korea), and sequencing electropherograms were analysed using Sequencher v.5.4.6. Primers used to sequence each product are listed in Table S1.

### 2.8. Minor Intron Sequence Conservation and SpliceAI Score Analysis

The locations of all human minor introns were retrieved from the Minor Intron Database [12] (https://midb.pnb.uconn.edu/), and the corresponding donor and acceptor nucleotide sequences were retrieved from the human reference sequence (GRCh38) using the bedtools ‘get fasta’ tool [23]. Sequence consensus motifs were plotted using WebLogo (https://weblogo.berkeley.edu/logo.cgi). We retrieved precomputed SpliceAI delta scores (https://basespace.illumina.com/s/otSPW8hnhaZR) [16] for all possible single nucleotide variants, retaining only scores that correspond to the minor intron splice site. Variants with a lower probability of representing a donor or acceptor motif were shown as negative values, and NULL was used if a variant did not impact splice site strength.

## 3. Results

### 3.1. Clinical Evaluation of the Family

The female proband (II:2) experienced a cardiac arrest at age 27 and a recurrent arrest at age 30 (Figure S2A). While her initial cardiac investigations were unremarkable, at the time of her second cardiac arrest, she had developed low ejection fraction without evidence of late gadolinium enhancement and significant conduction disease with left bundle branch block. She received an implantable cardioverter defibrillator with cardiac resynchronisation therapy. However, she went on to develop frequent and recurrent ventricular fibrillation refractory to antiarrhythmic therapy, including quinidine, with up to three appropriate shocks per month. An invasive electrophysiology study mapping of both the endocardial and epicardial surfaces identified abnormal potentials originating from the outer right ventricular outflow tract. Following a successful targeted radiofrequency ablation [24], she has not had a recurrent shock while off antiarrhythmic medications for 6 years with ongoing follow‐up.

The proband′s brother (II:1) was initially referred years prior to the proband′s arrest after an incidental Type 2 Brugada electrocardiogram pattern, and a procainamide challenge later revealed a Type 1 Brugada pattern, consistent with inducible BrS. He declined genetic testing at the time. Their father (I:1) has trifascicular block and preserved ejection fraction while their mother (I:2) is clinically well. There was no additional significant family history (Figure S2A).

### 3.2. Cardiac Genetic Testing

Exome‐based testing of a 184‐gene comprehensive cardiology panel in the proband identified a novel *SCN5A* VUS, NM_000335.5:c.392+3A>G in the donor splice site of minor Intron 3 (ClinVar variant ID # 918002). No other clinically relevant variants were reported. This variant is absent from gnomAD population controls, and in silico splice prediction tools, MaxEntScan, AdaBoost and random forest did not predict disrupted splicing, as the native minor intron splice site was not recognised. SpliceAI scored a donor loss *Δ* score of 0.62, which is above the threshold of 0.5 for moderate to high confidence splice‐disrupting variants (Table S3). The variant segregated with disease in the proband′s affected brother and father and was absent in her unaffected mother (Figure S2B).

### 3.3. *SCN5A* c.392+3A>G RNA Splicing Analysis

**Figure 1:**
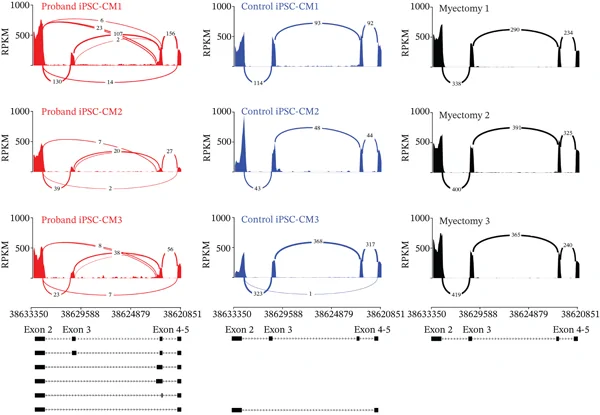
Sashimi plots of *SCN5A* splicing across Exons 2–5 in induced pluripotent stem cell cardiomyocytes treated with cycloheximide and myectomy controls. Panels show sashimi plots from cycloheximide‐treated iPSC‐CM lines derived in triplicate from the proband (red) and three unrelated control lines (blue) and three female control myectomy samples (black). Loops with numbers represent sequencing reads supporting each splice junction. Lower panel shows detected transcript isoforms across Exons 2–5. RPKM, reads per thousand mapped reads.

To assess the functional consequences of the *SCN5A* c.392+3A>G variant, we analysed RNA splicing in patient‐derived tissues. RNA extracted from peripheral blood of the proband and controls did not reliably support amplification of *SCN5A* transcripts, likely due to low expression levels in blood (not shown). We therefore derived iPSC‐CMs from the proband′s peripheral blood mononuclear cells, in triplicate, to serve as surrogate patient heart tissue. Transcriptome sequencing was performed on RNA extracted from iPSC‐CMs cultured in triplicate, with or without cycloheximide to inhibit nonsense‐mediated decay and enable detection of transcripts potentially degraded by this pathway, from the proband and three unrelated female control iPSC‐CMs, and three myectomy samples from unrelated females. Canonical *SCN5A* splicing was observed in all samples, as would be expected from the wild‐type allele. However, the proband′s iPSC‐CMs showed multiple additional spliced *SCN5A* transcripts flanking Intron 3, with and without cycloheximide, that were not detected with transcriptome sequencing of control iPSC‐CMs or myectomy samples (Figure 1 and Figure S3, respectively). These transcripts had the following splice junctions: Exon 3 skipping with a 58 bp 5 ^′^ truncation of Exon 4, skipping of Exons 3 and 4, Exon 3 skipping with a 96 bp or 117 bp 5 ^′^ extension of Exon 4 and a 27 bp 3 ^′^ extension of Exon 3 with a 1 bp 5 ^′^ deletion of Exon 4 (Figure 2). Four encode a frameshift and premature termination codon, while one encodes an in‐frame truncation of 59 amino acids, p.Thr92_Gln150del (Table S4). All alternatively spliced transcripts were confirmed in patient‐derived iPSC‐CMs by RT‐PCR and sequencing T/A cloned products. The transcript showing Exon 3 skipping with a 96 bp extension of Exon 4, which was absent in transcriptome sequences of control iPSC‐CMs and myectomy tissue, was amplified with RT‐PCR in the control samples (Figure 2). This isoform is absent in over 300,000 transcriptomes in the SpliceVault database [25], and it may be present at trace levels, or represent an RT‐PCR amplification artefact, in our controls. When analysing patient‐derived iPSC‐CM transcriptome sequencing reads spanning unique exon junctions in a semiquantitative analysis, approximately 80% span frameshifting transcripts and 20% span the transcript encoding an in‐frame deletion (Table S5). These estimates include the RT‐PCR control‐detected isoform since this was readily detected with transcriptome sequencing of patient‐derived iPSC‐CMs but was absent in transcriptome sequence reads of control iPSC‐CMs and myectomy tissue.

Figure 2
*SCN5A* c.392+3A>G induced alternative splicing. Left panels depict alternative splicing in patient‐specific iPSC‐CMs across *SCN5A* Exons 2–5 (numbered boxes) with intronic splice dinucleotide motifs. Sanger verification is shown above each novel mRNA junction. Right panels show RT‐PCR amplification products using RNA extracted from three patient‐specific iPSC‐CM lines and two control iPSC‐CM lines, treated with DMSO or cycloheximide, and three myectomy samples. (A–C) Forward primer anneals to Exon 2, (D) Exon 2 and 96 bp retention of Intron 3, (E) Exon 2 and 117 bp retention of Intron 3 or (F) 27 bp extension of Exon 3. cyclo, cycloheximide.

**Figure:**
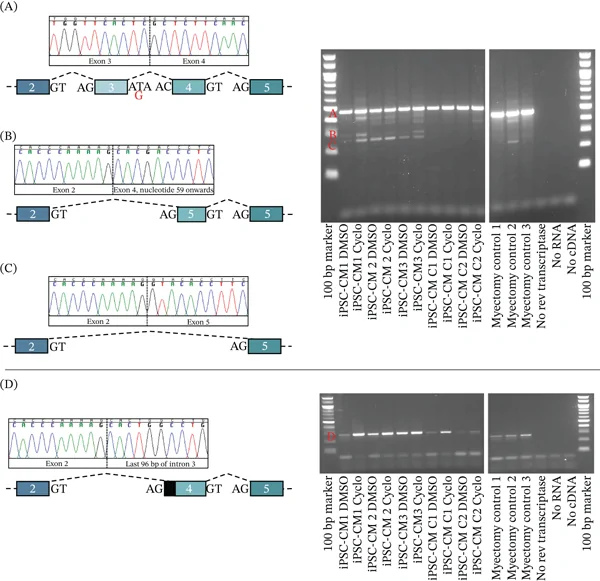

**Figure:**
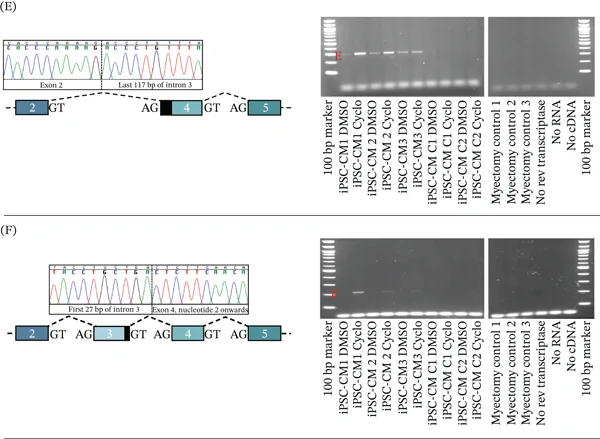

We reclassified the *SCN5A* c.392+3A>G variant pathogenicity using the ACMG/AMP variant classification framework [26] and PVS1 decision tree, as per the ClinGen Sequence Variant Interpretation Splicing Subgroup [27]. The variant was initially classified as a VUS as it was absent from control population data (PM2) with a SpliceAI *Δ* score ≥ 0.2 (PP3). We interpreted the mixed splicing outcomes using the PVS1 decision tree, as per the ClinGen Sequence Variant Interpretation Splicing Subgroup. Frameshifting transcripts use a cryptic splice motif that disrupts the reading frame and is predicted to undergo nonsense‐mediated decay in the loss‐of‐function intolerant *SCN5A* gene (pLi = 0.91; o/e = 0.21) and support PVS1 classification. The transcripts with an in‐frame deletion remove Amino Acids 92–150 in a transmembrane domain that is critical to SCN5A function, and a comparable in‐frame deletion of Residues 132–161 produced a nonfunctional protein [28]. Therefore, these in‐frame transcripts also support PVS1 classification. Finally, canonical splicing is accounted for by the heterozygous wild‐type allele in iPSC‐CMs and does not require classification. Therefore, applying PM2 with PVS1 upgrades the VUS to likely pathogenic.

### 3.4. Characterisation of Novel Splice Junctions

Inspection of the novel splice junctions revealed sequences matching the consensus motifs of both minor and major introns (Figure S4). One transcript removes Intron 3 using a cryptic minor intron donor site paired with an atypical CG dinucleotide acceptor site located one base downstream of the canonical minor intron acceptor site and is consistent with minor spliceosome use. This transcript encodes a frameshift and has trace expression levels, as it was only detected in cycloheximide‐treated patient iPSC‐CMs using a sequence‐specific PCR primer (Figure 2F).

The remaining alternatively spliced transcripts use the canonical Exon 2 major intron donor site paired with novel cryptic major intron AG acceptor sites, suggesting splicing by the major spliceosome (Figure 2 and Figure S4). The novel splice site strengths, as determined by MaxEntScan, are listed in Table S4.

We searched for these alternative splice junctions in over 300,000 publicly available RNA sequencing samples using SpliceVault [25]. Only skipping of Exons 3 and 4 was reported in 421 (1%) samples. SpliceVault also reported Exons 3–5 and 3–6 skipping, and in three samples, use of a cryptic minor intron AT donor site 356 bp downstream of Exon 3, none of which were observed in our iPSC‐CMs.

### 3.5. Sequence Conservation and SpliceAI *Δ* Scores of Minor Introns

**Figure 3:**
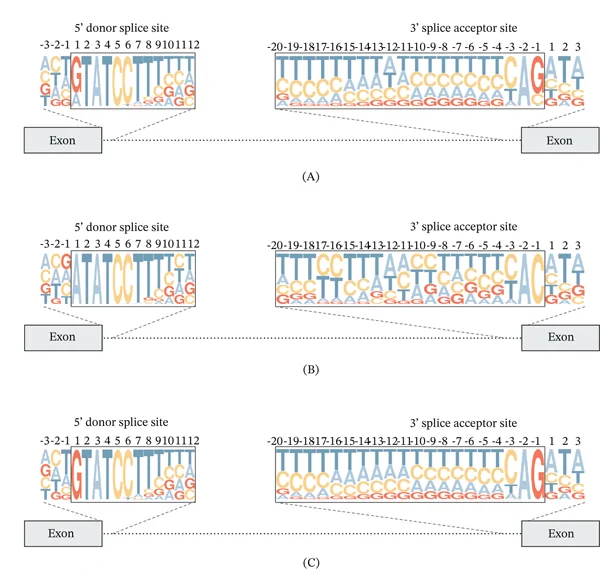
Minor intron splice site consensus sequences. (A) Consensus sequence of all human minor introns, *n* = 770. (B) AT‐AC type minor introns, *n* = 172. (C) GT‐AG type minor introns, *n* = 555. The height of each nucleotide corresponds to its frequency at that position.

To define the critical splice site nucleotides of minor introns, we retrieved the locations of all minor introns from human genes (*n* = 770) using the Minor Intron Database [12], and the corresponding nucleotide sequences (Table S6). The minor introns were grouped into the main AT‐AC (*n* = 172) or GT‐AG (*n* = 555) subtypes, and the consensus donor and acceptor sequence motifs were plotted (Figure 3 and Tables S7 and S8). These consensus donor and acceptor sequences were almost invariant across the first nine and last two intronic nucleotides, respectively, suggesting they are important for intron recognition.

**Figure 4:**
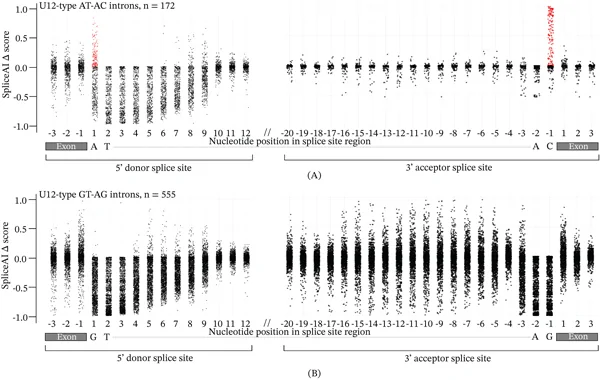
SpliceAI *Δ* scores for AT‐AC and GT‐AG minor introns. Each dot represents a SpliceAI *Δ* score for a single nucleotide variant′s impact on the splice donor (left panels) or acceptor site (right panels). Positive values—increased probability of functional splice site; negative values—reduced probability of functional splice site. (A) SpliceAI *Δ* scores across AT‐AC minor introns. Red dots represent A>G (donor site) or C>G (acceptor site) nucleotide substitutions. (B) SpliceAI *Δ* scores across GT‐AG minor introns.

We next plotted the SpliceAI *Δ* scores of all possible single nucleotide variants in these regions where positive and negative values correspond, respectively, to a higher or lower probability that the motif is a functional splice site (Figure 4 and Tables S9 and S10). Collectively, variants at each of the nine conserved donor residues show lower scores than the flanking nonconserved intronic residues (*p* < 0.0001), indicating a lower probability that the variants maintain a functional donor site. One exception is A>G variants at the first nucleotide of AT‐AC introns (shown in Figure 4A as red dots), which show higher SpliceAI scores, indicating a higher probability that these variants maintain a functional donor. For acceptor sites, few variants in AT‐AC introns were predicted to alter splice site probability, except for C>G variants at the last nucleotide of the intron (Figure 4A, red dots). In GT‐AG minor introns, variants located in the last three intronic nucleotides showed lower SpliceAI scores than those in other acceptor nucleotide positions, indicating a lower probability of a functional acceptor site (Figure 4B).

## 4. Discussion

We determined the clinical relevance of an *SCN5A* minor intron donor splice site variant in an individual with a personal and family history of cardiac arrhythmias. Three commonly used in silico splice prediction tools were uninformative for this variant, whereas SpliceAI predicted a donor loss. We derived iPSC‐CMs from the proband to assess splicing outcomes due to low expression of *SCN5A* in blood. Transcriptome sequencing revealed multiple alternatively spliced *SCN5A* transcripts, with most conforming to major intron GT‐AG splice site motifs, although one product involved splicing at minor intron motifs. Most alternatively spliced products were not detected in publicly available RNA sequencing datasets, highlighting the need for direct functional analysis of patient‐derived RNA. When examining all possible SpliceAI predictions across minor introns, the first nine nucleotides of the donor site frequently predict splice disruption, with implications for prioritising variants in these positions for functional assessment. In contrast, relatively few variants were predicted to disrupt splicing in the ultraconserved minor intron AT‐AC acceptor dinucleotides, suggesting an area for improvement in splice prediction tools. Based on our RNA analyses, the *SCN5A* VUS was reclassified as likely pathogenic, with important implications for ongoing clinical management of the family.

### 4.1. Clinical and Genetic Significance of the SCN5A c.392+3A>G Variant

Our findings support reclassification of the *SCN5A* variant as likely pathogenic, consistent with a loss‐of‐function mechanism that would explain the cardiomyopathy and arrhythmogenic phenotypes observed in the family. The novel variant segregates with BrS, conduction disease and cardiomyopathy across two informative meioses in the family. Two frameworks for scoring segregations are the ClinGen Cardiomyopathy Variant Curation Expert Panel specifications [29], which require at least three meioses for PP1_Supporting, and a recent Bayesian framework [30], which would support PP1_Moderate with two segregations. Since ClinGen *SCN5A* or arrhythmia specifications are not yet available, we follow the established cardiomyopathy rules and so did not apply PP1.

RNA analysis of patient‐derived iPSC‐CMs revealed four variant‐associated cryptic products plus an isoform that was absent in control iPSC‐CMs and myectomy transcriptome data but detected in these samples with RT‐PCR. Four of these transcripts encode a premature stop codon, and one encodes an in‐frame deletion. While multiple factors influence the alignment and read depth of transcriptome reads spanning novel exon junctions, there were more sequencing reads spanning novel exon junctions of transcripts encoding premature termination codons than the in‐frame deletion. Disruption of *SCN5A* splicing leading to in‐frame deletion or frameshift variants with loss‐of‐function effects has previously been reported to underlie BrS [31, 32]. Transcripts encoding a premature termination codon will likely undergo nonsense‐mediated degradation and lead to loss of function via haploinsufficiency. The in‐frame deletion removes amino acid residues p.Thr92_Gln150del from the functionally critical first transmembrane region of SCN5A and has not been previously reported. However, a comparable in‐frame deletion of Exon 4 encoding transmembrane region Amino Acids 132–161 was reported in an individual with BrS, and expression of an Exon 4 deletion construct in HEK293 cells was nonfunctional [28]. Therefore, the combined mechanism of the four variant‐associated cryptic products plus an isoform that was absent in control iPSC‐CMs and myectomy transcriptome data but detected in these samples with RT‐PCR is one of loss of function through haploinsufficiency and a possible dominant‐negative protein that remains to be experimentally confirmed. It is plausible that the proband′s father and brother express different levels or combinations of cryptically spliced transcripts and thus contributing to the variable expressivity in their clinical phenotypes.

Since the variant occurred at the third nucleotide of the intron, we could not apply PVS1 directly from the ACMG classification guidelines [26]; as for splicing variants, this only applies to the first and last two nucleotides of the intron. Instead, we required direct functional assessment of the impact on splicing using the iPSC‐CMs from the patient, as blood RNA did not support amplification of *SCN5A* transcripts. The variant results in an aberrant splicing profile that we have interpreted as loss of function from semiquantitative analysis of novel junction‐spanning reads in an assay conducted using patient‐derived tissue. Four isoforms, represented by 80% of novel junctional reads, encode a frameshift and premature termination codon. Another isoform, represented by 20% of novel junctional reads, encodes an in‐frame deletion of unknown effect, although a comparable in‐frame deletion was shown to be nonfunctional [28]. These data allowed us to interpret the mixed splicing results using the ClinGen SVI splicing subgroup guidelines. Canonical splicing is derived from the heterozygous wild‐type allele. We assign PVS1 to frameshifting transcripts as they use cryptic splice site motifs that disrupt the reading frame and predict nonsense‐mediated decay. We also assign PVS1 to in‐frame transcripts as they remove a transmembrane region that is critical to protein function. Therefore, applying the same classification to protein‐disrupting isoforms enabled reclassification to likely pathogenic. This clinically meaningful reclassification will enable the variant to be used in predictive testing of additional family members and enable future reproductive options.

### 4.2. Broader Implications for Variant Interpretation

Our analyses of minor intron consensus sequences and SpliceAI scores have broader implications for variant interpretation. The ACMG/AMP produced a widely adopted framework for the classification of variant pathogenicity [26]. In these guidelines, the PVS1 criterion is applied for splice donor and acceptor variants in canonical ±1 or 2 splice site dinucleotides, where exon skipping predicts a frameshift, in a gene where loss of function is an established disease mechanism. Further recommendations were issued on how to classify variants that impact splicing or result in a loss of function [27, 33]. The ClinGen SVI splicing subgroup defined a splice region variant as occurring within the region of the donor motif (last 3 bases of the exon and 3–6 nucleotides of intronic sequence adjacent to the exon), or acceptor motif (first base of the exon and 3–20 nucleotides upstream of the exon boundary) [27]. These definitions are relevant to U2 major introns; however, our analyses indicate the first nine nucleotides of U12 donor sites are both conserved and enriched for predicted splice‐disrupting variants. To improve prioritisation of minor intron splice donor variants for functional validation, more emphasis should be given to variants in these nine residues than ACMG/AMP guidelines currently recommend.

We further recommend that variants in the first and last two nucleotides of a minor intron should be evaluated for the potential to disrupt splicing by considering donor–acceptor dinucleotide pairs, as some combinations may be incompatible with splicing. For example, if a U12‐type dinucleotide pair AT‐AC changes to GT‐AC or AT‐AG, a positive Splice AI score may be incorrect, as these pairings do not occur or are very rare among U12‐type human introns. We have provided the counts of donor and acceptor pairings in the minor intron database to guide such assessment of motif compatibility (Table 1).

**Table 1:** Counts of minor intron donor–acceptor splice dinucleotide pairs.

Splice donor–acceptor dinucleotide pair	Minor intron database count
AT‐AA	6
AT‐AC	172
AT‐CC	1
AT‐AG	15
AT‐TG	1
AT‐AT	9
GT‐AG	555
GT‐GG	6
GT‐TG	1
GT‐AT	4

### 4.3. Novelty and Mechanistic Insights

Our discovery of four variant‐associated cryptic products plus an isoform that was absent in control iPSC‐CMs and myectomy transcriptome data but detected in these samples with RT‐PCR that arise from a minor intron variant offers new insight into how combinations of donor and acceptor pairings disrupt splicing. The *SCN5A* variant redirected splicing predominantly to cryptic major spliceosome sites, rather than combinations of minor and major spliceosomes. It has been postulated that major and minor spliceosomes can cooperate to enhance splicing of adjacent major and minor introns or they may compete for access to introns recognised by both complexes [13]. This cooperative splicing may explain why disruption of a minor intron redirects alternative splicing to minor and major splice sites [34], with a bias towards the more common major spliceosome pathway. Consistent with this model, a minor intron splice site variant in *LKB1* generated multiple alternative transcripts, and selective inhibition of the major or minor spliceosome, or both, revealed which spliceosome directed each event [35].

In our study, disruption of the *SCN5A* minor intron donor site produced four variant‐associated cryptic products plus an isoform that was absent in control iPSC‐CMs and myectomy transcriptome data but detected in these samples with RT‐PCR, of which four used major spliceosome sites. Cycloheximide treatment of patient‐specific iPSC‐CMs revealed an additional low‐abundance transcript that used an atypical minor intron site. By analysing the splice site motifs of these alternative transcripts, we speculated as to whether the major or minor spliceosome was most likely responsible for each event. This could be further explored with expression of an *SCN5A* minigene construct containing the c.392+3A>G variant and cotransfection of antisense oligonucleotides directed at U2 or U12 small nuclear RNAs. Inhibition of U2 or U12‐spliceosomes would reveal which alternatively spliced products are impacted. Mechanistic insights from such studies could improve the prediction of alternative splicing outcomes for minor intron variants.

Our analysis of patient RNA and SpliceAI scores highlights the potential for minor intron variants to be misclassified or overlooked due to the limitations of in silico tools and blood‐based RNA testing of cardiac genes. In silico tools show moderate accuracy in identifying splice‐disrupting variants but are less effective at predicting alternative splicing outcomes. SpliceAI did not predict splice disruption for many variants in the conserved AC acceptor dinucleotides of AT‐AC introns, and such variants may not be prioritised in clinical workflows. We have previously highlighted the value of using fresh blood RNA to functionally assess the impact of candidate splice‐disrupting variants across a range of cardiac disease genes [21, 36]. While amplification of *SCN5A* transcripts is possible using fresh blood RNA, the expression level of this gene is much higher in iPSC‐CMs and myectomy tissue, and hence, these tissues detect a bigger dynamic range of alternatively spliced transcripts [21]. This motivated us to use patient‐derived iPSC‐CMs to analyse the splicing outcomes of the *SCN5A* c.392+3A>G variant. Most alternative splicing junctions were not observed in SpliceVault and would not have been predicted a priori, highlighting the value of direct confirmation of alternative splicing in a disease‐relevant tissue. Our mapping of SpliceAI scores across minor intron splice sites will be a useful resource for prioritising minor intron variants in other genetically unexplained Mendelian diseases.

Our study is not without limitations. Only three family members were available for segregation of the *SCN5A* variant. iPSC‐CMs are a surrogate heart tissue model that does not fully replicate adult cardiac tissue, and some low‐level splice isoforms may be artefactual or not present in vivo. RNA expression levels and splicing events in 2‐D iPSC‐CM cultures at Day 30 of differentiation, as we have used, most closely resemble 8‐ to 10‐week‐old prenatal human heart samples [37]. During development of the human heart, there is a switch in *SCN5A* isoform whereby a ‘fetal’ Exon 6 is replaced by an alternative ‘adult’ Exon 6; however, there is no evidence of developmental regulation of *SCN5A* minor intron splicing, including in matured iPSC‐CMs [38]. Whether iPSC‐CMs replicate cryptic splicing in the adult heart would require primary heart tissue from a variant carrier in the family, which was not available. Alternatively, given the conservation of minor intron sequence motifs across species, an *SCN5A* variant knock‐in animal model may provide suitable primary heart tissue to evaluate RNA splicing outcomes and allow investigation of cardiac electrophysiology and potential therapeutic interventions.

## 5. Conclusions

Our study provides new insights into the functional impact of a minor intron variant in *SCN5A* and highlights the value of patient‐derived iPSC‐CMs in variant interpretation. Future work should explore the clinical relevance of variants in the first nine nucleotides of U12‐type intron donor splice sites and develop improvements in in silico modelling of minor intron variants to predict impact on splice site strength.

## Author Contributions

Conceptualisation: Emma S. Singer, Zachary Laksman and Richard D. Bagnall. Funding acquisition: Richard D. Bagnall. Methodology: Emma S. Singer and Richard D. Bagnall. Data curation: Emma S. Singer, Serena Li, Ginell N. Ranpura and Seakcheng Lim. Formal analysis: Emma S. Singer, Sandra T. Cooper and Richard D. Bagnall. Resources: Sean Lal, Zachary Laksman and Richard D. Bagnall. Writing—original draft: Emma S. Singer and Richard D. Bagnall. Review, editing and final approval: all authors.

## Funding

This work was supported by an NSW Health Cardiovascular Senior Scientist Grant to RDB.

## Ethics Statement

Written informed consent was provided by participants enrolled in protocol H19‐01941 approved by the University of British Columbia Ethics Committee, Canada. Control participants were enrolled in protocols X19‐0108 and 2019/ETH00461 for induced pluripotent stem cell (iPSC) studies, as approved by the Sydney Local Health District Ethics Review Committee, Australia.

## Conflicts of Interest

The authors declare no conflicts of interest.

## Supporting Information

Additional supporting information can be found online in the Supporting Information section.

## Supporting information


**Supporting Information 1** Figure S1. Consensus sequences of U2‐type and U12‐type introns. Figure S2. Family pedigree and *SCN5A* c.392+3A>G genotyping. Figure S3. Sashimi plots of *SCN5A* splicing across Exons 2–5 in induced pluripotent stem cell cardiomyocytes treated with DMSO. Figure S4. Donor and acceptor splice sites of cryptic splice products.

**Supporting Information 2** Table S1. Primers used in this study. Table S2. Details of patient‐specific induced pluripotent stem cell cardiomyocytes and myectomy samples. Table S3. Population frequencies and splice in silico prediction scores for SCN5A c.392+3A>G. Table S4. Predicted outcomes for each alternatively splice product detected. Table S5. Transcriptome read counts spanning novel splice junctions. Table S6. Location and nucleotide sequences of minor introns in human genes (*n* = 770). Table S7. Location and nucleotide sequences of AT‐AC minor introns in human genes (*n* = 172). Table S8. Location and nucleotide sequences of GT‐AG minor introns in human genes (*n* = 555). Table S9. AT‐AC minor intron splice site SpliceAI scores. Table S10 GT‐AG minor intron splice site SpliceAI scores.

## Data Availability

The data that support the findings of this study are available in the Supporting Information of this article.
