# Supplementary material for: SCN5A U12‐Type Minor Intron Variant Causes Cryptic Splicing and Cardiac Arrhythmia Disease
Source: Hum Mutat. 2026 Sep 24;2026:4537601. doi: 10.1155/humu/4537601 (PMC13602164; doi:10.1155/humu/4537601)
Supplement: Supplementary file 1 [file HUMU-2026-4537601-s001.docx]

**SUPPLEMENTARY FIGURES**

**Supplementary Figure S1. Consensus sequences of U2-type and U12-type introns.** Consensus sequences of the 5’ donor, 3’ acceptor, and branch point sequence of **(A)** U2-type major introns and **(B)** U12-type minor introns. Exons are in boxes, introns are denoted by the dotted line, essential dinucleotides are in red, branch point adenosine is in purple.

**Supplementary Figure S2. Family pedigree and *SCN5A* c.392+3A>G genotyping. (A)** Family pedigree. Females are circles, males are squares; proband is indicated by an arrowhead; heterozygous (+/-) or genotype negative (-/-) for *SCN5A* c.392+3A>G. **(B)** Sanger confirmation of *SCN5A* c.392+3A>G genotype is indicated by the arrow.

**Supplementary Figure S3. Sashimi plots of *SCN5A* splicing across exons 2 to 5 in induced pluripotent stem cell cardiomyocytes treated with DMSO.** Panels show sashimi plots from DMSO-treated iPSC-CMs derived in triplicate from the proband (red) and three unrelated controls (blue). Loops with numbers represent sequencing reads supporting each splice junction. Lower panel shows detected transcript isoforms across exons 2 to 5. RPKM – reads per thousand mapped reads.

**Supplementary Figure S4. Donor and acceptor splice sites of cryptic splice products.** Donor and acceptor splice sites for each cryptic splice product is shown based on Sanger sequencing results grouped by splice sites that resemble either U12-type minor introns (top panel) or U2-type major introns (bottom panel). Exons are in boxes and are separated by introns with a vertical line (|), potential branch site is underlined with branch point adenosine bolded in purple.
